# Spatial and Temporal Patterns of Dengue Transmission along a Red Sea Coastline: A Longitudinal Entomological and Serological Survey in Port Sudan City

**DOI:** 10.1371/journal.pntd.0001821

**Published:** 2012-09-27

**Authors:** Osama M. E. Seidahmed, Safa A. Hassan, Mohamed A. Soghaier, Hanna A. M. Siam, Fayez T. A. Ahmed, Mubarak M. Elkarsany, Suad M. Sulaiman

**Affiliations:** 1 Department of Medical Entomology, National Health Laboratory, Khartoum, Sudan; 2 Public Health Laboratory, Red Sea State Ministry of Health, Port Sudan, Sudan; 3 National Control Program of Epidemics and Zoonotic Diseases, Khartoum, Sudan; 4 Nile College, Khartoum, Sudan; Centers for Disease Control and Prevention, United States of America

## Abstract

**Background:**

Dengue is an emerging health problem in several coastlines along the Red Sea. The objective of the present work is to elucidate spatial and temporal patterns of dengue transmission in Port Sudan.

**Methods/Findings:**

A longitudinal study with three cross-sectional surveys was carried out in upper, middle and lower class neighborhoods, from November 2008 to October 2009. Monthly, entomological surveys were followed by serological surveys in dengue vector-positive houses. Meteorological records were obtained from two weather stations in the city during the same time. Overall, 2825 houses were inspected. *Aedes aegypti* represented 65% (35,714/54,944) and 68% (2526/3715) of the collected larvae and pupae, respectively. Out of 4640 drinking water containers, 2297 were positive for *Ae. aegypti*. Clay-pots “Zeirr” followed by plastic barrels were key productive containers for pupae of dengue vector, 63% (n = 3959) and 26% (n = 1651), respectively. A total of 791 blood samples were tested using PanBio Capture/Indirect IgM ELISA. Overall, the sero-prevalence rate of dengue ranged between 3%–8% (41/791), compared to an incidence of 29–40 new cases per 10,000 (193/54886) in the same examined population. Lower and middle class neighborhoods had higher entomological indices compared with upper class ones (p<0.001). Although, dengue incidence rate was significantly lower in the middle and lower class neighborhoods (F = 73.97, d.f. = 2, p<0.001), no difference in IgM prevalence was shown. The city is subject to two transmission peaks in the winter (i.e. November–January), and summer (i.e. June–August). The serological peaks of dengue are preceded by entomological peaks that occur before the onset of winter (November) and summer (March) respectively.

**Conclusion:**

Dengue incidence is heterogeneously distributed across the neighborhoods of Port Sudan and exhibits a bi-cyclic intra-annual pattern. Hence, it should be feasible to carry out timely vector control measures to prevent or reduce dengue transmission.

## Author Summary

Dengue is a tropical infectious disease that is of emerging global importance. As a dengue vaccine is still a distant prospect, descriptive epidemiological studies are a vital tool for developing a surveillance system capable of preventing dengue outbreaks. In the current work, the investigators describe epidemiology of dengue in Port Sudan on the Red Sea. There, the disease has distinctive spatial and temporal patterns. Households use water storage containers to supplement Port Sudan’s poor public water supply. These containers provide breeding sites for dengue mosquitoes. Differences in water consumption patterns across neighborhoods result in differences in disease incidence. Our results suggest that Lower class neighborhoods may suffer dengue more than upper class ones. In addition, two transmission peaks are observed during the winter and summer seasons. These spatial and temporal patterns of dengue may describe dengue epidemiology along similarly affected coastlines of the Red Sea.

## Introduction

Dengue fever is the most important mosquito-borne viral disease in the world. The incidence of dengue has increased over ten-fold over the last three decades with an estimated 50 million cases in over 100 countries [1], [2]. The global emergence of dengue is linked to increasing travel of viremic people as well as dispersal of its main vectors (i.e. *Aedes aegypti* and *Ae. albopictus*) into new locations [3]–[5]. Climate variability and unplanned urbanization may contribute to dengue epidemics [6]–[8].

The Red Sea is a semi-enclosed Mediterranean sea surrounded by the African and Eurasian continents. It has a surface area of 438,000 km^2^ and water volume of 233,000 km^3^ that is linked to the Indian Ocean by a very shallow sill. Although dengue is reported from several surrounding countries such as Saudi Arabia, Yemen, Djibouti, Somalia and Sudan [9]–[16], little is known about dengue epidemiology along coastlines of the Red Sea.

Port Sudan city is Sudan's main seaport on the Red Sea. The main dengue vector *Ae. aegypti* has been reported in the area since the 1930s [17]. The dengue virus serotypes DEN1 and DEN2 were first detected in the 1980s in Port Sudan, while DEN-3 was recently identified in an outbreak [14], [16]. During the last two decades, the city has been subject to a number of dengue outbreaks *(Ministry of health, personnel communications)*.

In the present study, entomological and serological surveys in upper, middle and lower class neighborhoods of Port Sudan were coupled with meteorological parameters. The main objective of the present study was to elucidate spatial and temporal patterns of dengue transmission in Port Sudan city.

## Methods

### Ethics statement

Ethical approval for the study was granted by the Ethical Review Committee of the Ministry of Health, Sudan (2008). The objectives and procedures of the study were explained to the local health authorities, medical assistants and householders at each study site. Informed consent was obtained from all participants in accordance with the ethical standards of the Sudan committee.

### Study design

This was a descriptive stratified longitudinal study in upper, middle and lower class neighborhoods. The first survey was launched in October 2008 and the last one finished in October 2009. Monthly, two types of surveys were carried out: entomological pupal/demographic surveysand household serological surveys.

### Study area

Port Sudan (19 58 N, 37 21 E), is about 300,000 km^2^ with an estimated population of 450,000 people. Port Sudan has a humid Mediterranean climate and a service-based economy linked to shipping operations and trade. In addition, there are no suburbs or surrounding villages so food is transported to the city from other regions of Sudan and neighboring countries. Port Sudan is administratively divided into three sectors (Eastern, Middle and Southern) which are further divided into 39 neighborhoods.

### Study sites

**Figure 1:**
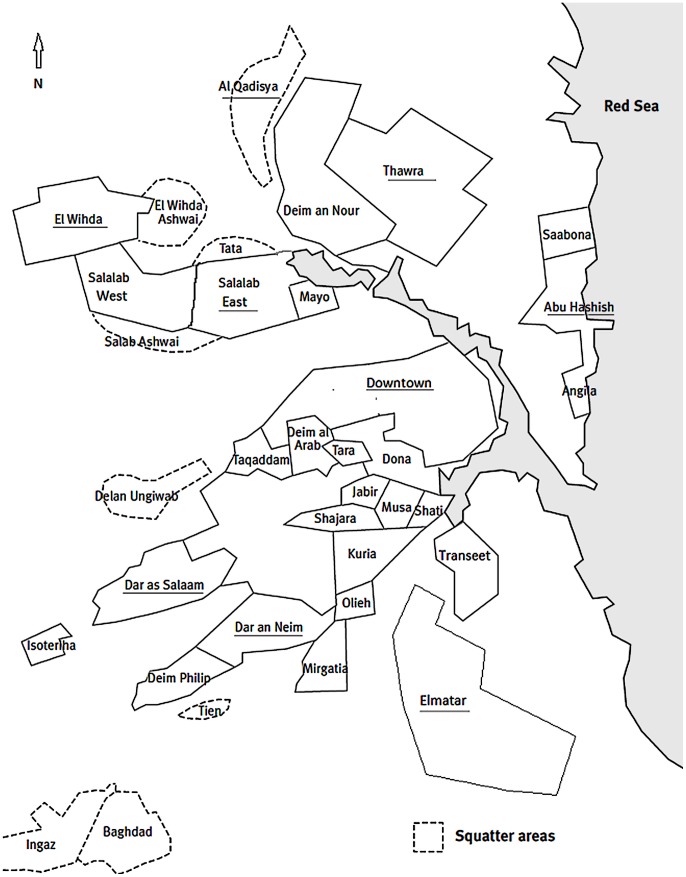
Port Sudan map. Sketch map of Port Sudan city shows approximate location of the study neighborhoods.

Nine residential neighborhoods were selected because they are a good representation of the city by class. We grouped these neighborhoods using indicators of living conditions such as method of water supply, on-site sanitation, and building material of houses into three strata: upper (Abuhasheish, Downtown and Elthora), middle (Elmatar, Salalab East, and Dar Elnaeem), and lower class neighborhoods (Dar Elsalam, Elgadisia and Elwihda).Approximate locations of the study sites are depicted in the map of the city (Figure 1).

### Meteorological data

Average monthly records from November 2008 to October 2009 were obtained from the meteorological authority. Meteorological parameters (minimum and maximum temperature, relative humidity, precipitation and, evaporation rates, wind speed and direction) were recorded on daily basis in two stations in Port Sudan.

### Entomological pupal/demographic survey

#### Sample size

A preliminary survey was carried out to determine the required sample size following the methods of Focks and Alexander [18]. A dispersion index was used where number of surveyed houses per demographic area is a function of key containers -productive to >70% of *Aedes* pupae- in the area. Accordingly, a dispersion index of one, two, three or above key container implies 10, 25, 50 or 100 houses require inspection, respectively. Since clay-pots and barrels were found to contain >70% of the pupae, 25–30 houses were inspected monthly in each neighborhood. Households were selected for inspection using a random number table and neighborhood sketch-maps. Inspected houses were separated by >300 meters in order to avoid duplicate representation of the same household cluster (i.e. the flight range of *Ae. aegypti)*.

#### Procedures

Monthly, a well-trained team composed of six health workers and two supervisors visited the houses. The team inspected water-containers for aquatic stages of mosquitoes. Numbers of containers, ‘wet’ containers and containers that harbored pupae and/or larvae of mosquitoes were counted. Moreover, the numbers of pupae and larvae [large (4^th^/3^rd^ instars) and small (1^st^/2^nd^ instars)] were counted, by container location (indoor/outdoor), type (material of the container) and volume (depth×area). Also, information was collected for positive covered containers and type of water supply (donkey transporters/pipeline/motorized transporters). In addition, proximity of positive containers to shading and presence of organic material were recorded. All detected pupae and larvae of mosquitoes were transferred into labeled vials, transferred to the field insectary in Port Sudan where the laboratory work was completed. The number of occupants in each screened house (as the number of people who slept in the house the previous night) at the time of each survey was recorded.

#### Mosquito rearing and identification

The contents of vials were transferred separately to small cups covered with a netting material and secured with a rubber band; these were held in the field laboratory until adult emergence occurred. Where appropriate, specimens of mosquitoes were identified as emerged adults, larvae and pupae using taxonomic keys [19]–[21].

### Household serological survey

A random stratified sampling strategy was followed.

#### Sample size

The sample size was determined using OpenEpi 2.2 software [*n* = [DEFF*Np(1−p)]/[(d^2^/Z^2^_1−α/2_*(N−1)+p*(1−p)]. The input criteria were the number of surveys and the number of strata (upper, middle and lower class neighborhoods). Members of those households positive for *Aedes* mosquitoes were recruited. During surveys, residents with symptoms of dengue (i.e. high fever and two of the following criteria: rash, severe headache, severe eye pain, joint pain, and muscle and/or bone pain) were referred to the hospital but excluded from the sample size.

#### Blood sampling

Monthly, one household member was randomly recruited for blood sampling per inspected household. After obtaining oral consent, 1 ml of venous blood was collected by the medical assistants in plain vials (Greiner, Minicollect). The serum was immediately isolated and stored in a sterile vial at −20°C in the regional Public Health Laboratory in Port Sudan. Then, serums were shipped to the Virology Laboratory - National Health Laboratories in Khartoum.

#### ELISA procedures

Kits of enzyme-linked immunosorbent assay (ELISA) were used to detect dengue-specific IgM antibodies in all samples according to the manufacturer's instructions (PanBio, Brisbane, Australia). Results were calculated as “Panbio Units” with results 9.0, 9.0–11.0, and ≥11.0 defined as negative, equivocal, and positive, respectively. Samples that initially scored as equivocal were retested to confirm the result.

### Retrospective survey on reported cases

All dengue cases during the study period were reviewed retrospectively. These cases were reported through the health information system vertically from health dispensaries and the main hospitals of Port Sudan up to the central level. Then, only cases whose home address was from the study neighborhoods were selected for the study. All clinical criteria and laboratory data from each case were checked further by an epidemiologist to confirm its accordance with the dengue case definition and management protocol of the Ministry of Health and WHO guidelines [1].

### Calculations and data analysis

Monthly, entomological indices were calculated for each study site. These include both *Stegomyia* indices:

House Index (HI) = percentage of houses or premises positive for *Aedes* aquatic stages, Container Index (CI) = percentage of water containers positive for *Aedes* aquatic stages, Breteau Index (BI) = number of positive containers per 100 houses in a specific location; Pupal indices (Pupal/Person (P/P) = total number of collected pupae/total number of inhabitants in the inspected households Pupal/children (P/C) = total number of collected pupae/total number of children under five years in the inspected households.

All the data analysis was performed using version 2.3 of OpenEpi software for Windows [22]. Comparison between two groups was done using a Chi square test. ANOVA was utilized to compare between the study's strata. Pearson correlation was performed to associate entomological, serological and meteorological data.

## Results

### General characteristics of study sites

A total of 2825 households were accessible and inspected in Port Sudan city. Average family size was larger in the upper class neighborhood (6.1) compared to the middle and lower strata (5.8 and 5.6, respectively). This was associated with greater consumption rate of drinking water in the high stratum (13 liters/person) compared to the other two strata(9 and 7 liters/person, respectively). Although donkey-drawn water tankers were the main method of water supply in Port Sudan (75%), this was only true in 51% of households in the upper class neighborhood due to the presence of public water pipes (33%) and motorized tankers (16%) in these areas (Table 1).

**Table 1:** Descriptive statistics of the neighborhood strata of Port Sudan during the study period: November 2008–October 2009.

Neighborhood stratum	Average Inspected Houses (N)	Household density (N)	child/house (N)	female/house (N)	Median Water consumptionχ	Median Water turnover*	%Method of water supply (range)ψ		
							Public pipeline	Motorized Tanker	Donkey-Tanker
**Upper Class**	25.33±1.12 (912)	6.06+2.49 (5527)	1.56+1.6 (1421)	3.8+2.22 (3,467)	13(11–16)	7(6.22+1.95)	33% (26–39)	16% (6–27)	51% (38–67)
**Middle Class**	26.44±0.56 (952)	5.81+2.32 (5532)	1.62+2.27 (1542)	3.68+2.0 (3,503)	9 (7–9)	6 (5.93+0.56)	19% (1–51)	2.9% (2–3)	78.5% (46–96)
**Lower Class**	26.69±0.13(961)	5.59+2.31 (5368)	1.6+1.59 (1538)	3.46+1.9 (3,328)	7 (5–8)	10 (10.56+3.08)	2% (0–5)	2.3% (1–4)	95.6% (93–99)
**Average (N)**	26.16±0.5 (2825)	5.81+2.37 (16,427)	1.59+1.85 (4,491)	3.65+2.08 (10,298)	9.5 (156,212)	7.7 (2825)	17.7% (499)	6.9% (196)	75.4% (2130)

χ Median drinking water consumption (in liters) per person per day in each stratum calculated from numbers of water containers during the inspection surveys; ranges of study neighborhoods are shown between parentheses.

* Median number of water turnovers per month (average numbers in parenthesis).

ψ Percentages are calculated per total of each row (stratum); ranges in parentheses show minimum to maximum between neighborhoods of each stratum.

### Meteorological data

While the mean minimum temperature (19.3°C) was recorded on March 2009, the mean maximum one (43.7°C) was on July 08. The minimum relative humidity was recorded on June 09 (30%), compared to the maximum (67%) on November 08. In addition, a short rainy season occurred in two months December 2008 (1.2 mm) and January 2009 (3 mm), with very little rainfall in November, February and July (<0.00001 mm). The highest wind speed occurred in January 2009 (1.3 knots) while the lowest one was in October 2009 (6 knots). The highest evaporation rate was on July 2009 (15.7 mm) while the lowest one was on November 2008 (6.4 mm).

### Key productive containers of dengue vector

**Figure 2:**
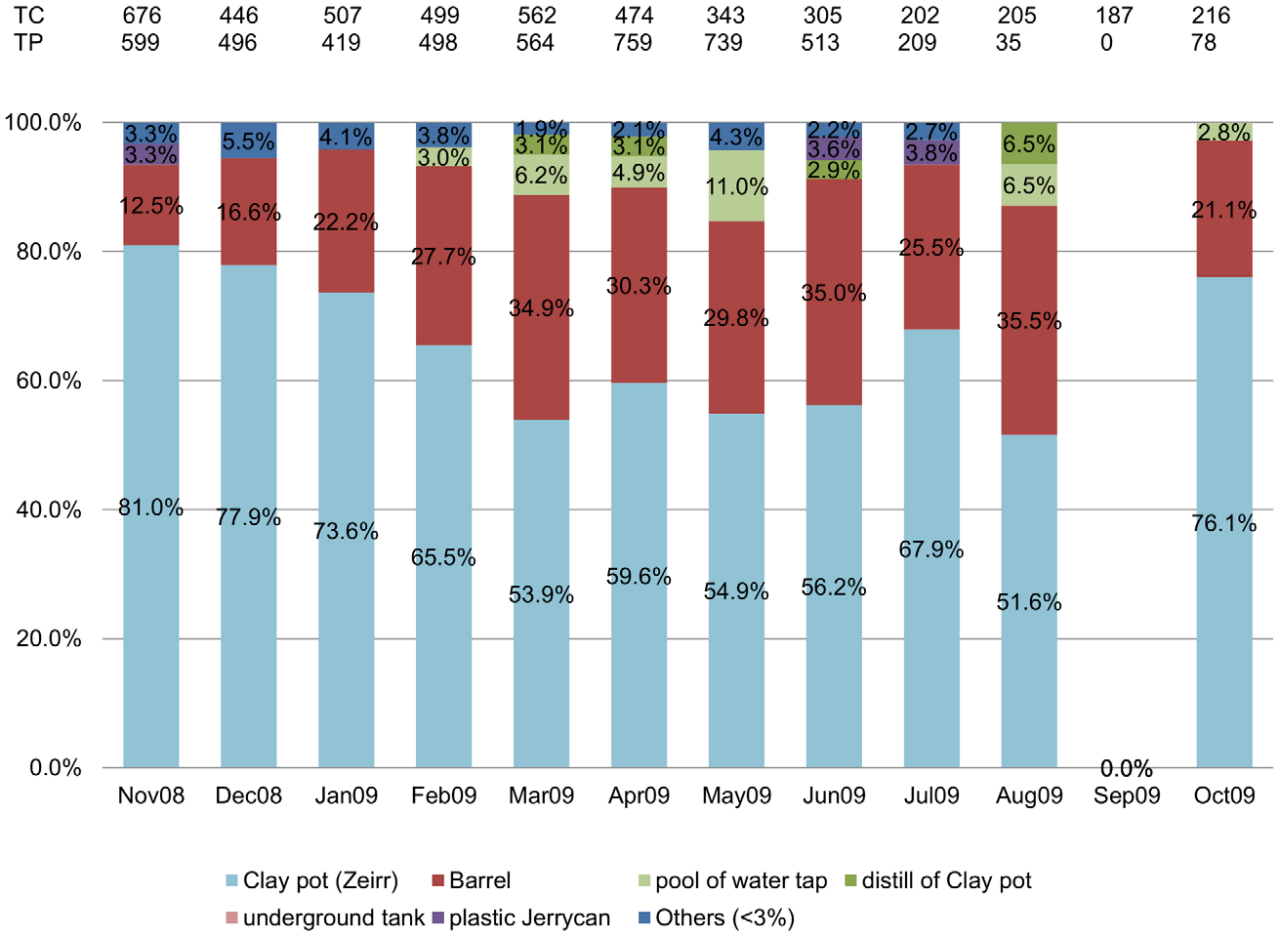
Types of productive containers of dengue vector. Clay pots and barrels are among the nine productive containers of *Aedes aegypti (L)* identified in Port Sudan during the study period (November 2008–October 2009).

A total of 2297 out of 4640 water containers (49.4%) were found positive for *Ae. aegypti*. However, over 70% of the positive containers were covered with lids, and 98% of these were located indoors. Clay-pots (in Arabic “Zeirr”) followed by plastic barrels represented the key breeding containers for pupae of *Aedes* mosquito, containing 63% (number of pupae = 3959) and 26% (number of pupae = 1651) of pupae, respectively. Other containers (representing <10%) included: pools of excess tap water, underground tanks, pans, pools filtered water from clay-pots, wells and plastic Jerry cans. The highest dispersion of *pupae* among container types was shown in May (*i.e.* six types found positive for *Aedes*), compared to the lowest in October (*i.e.* three types) (Figure 2).

### Species composition of container mosquitoes

A total of 54,944 larvae and 3715 pupae of mosquitoes were collected during the entomological surveys (Table 2). Morphological identification showed that *Ae. aegypti* constituted 65% and 68%, of the collected larvae and pupae, respectively. Fewer pupae and larvae of *Aedes aegypti* were collected in upper class neighborhoods (45% and 59%) compared to the middle (66% and 75%), and lower class (65% and 72%) ones, respectively. Other collected mosquitoes were identified as: *Culex quinquefasciatus* Say (18.5%), *Culex sitiens* Wiedemann (12%) and *Ae. caspius* Pallas (0.1%), and unidentified *Culex spp.* belonging to group IV (11%). The dengue vector was co-breeding with one of the above species in few numbers of mixed containers (1.68%).

**Table 2:** Numbers of collected mosquitoes during the study period Nov '08–October 2009.

Neighborhood strata	Number of containers		Total of collected larvae		Total of collected pupae	
	inspected	positive	All	*Ae. aegypti*	All	*Ae. aegypti*
**Upper class**	1401	579	18036	8233 (45%)	1217	724 (59%)
**Middle class**	1528	805	18840	11368 (66%)	1190	881(75%)
**Lower class**	1711	913	18068	11742 (65%)	1308	940 (72%)
**Grand total**	**4640**	**2297**	**54944**	**31343 (65%)**	**3715**	**2545 (68%)**

### IgM ELISA results

A total of 791 individual blood specimens was tested using PanBio Capture/Indirect IgM ELISA (Table 3). Participants ranged in age from 3 months to 80 years old; females represented 64% (506/791) of the study population. Dengue IgM high-titer serums were shown in 5.2% (41/791) specimens. There is a significant difference in IgM positive results between age groups :<5, 6–17, 18–39, 40–60 and >60 years (χ2 = 5.05, *d.f.* = 5, p = 0.03). Among these age groups, higher dengue attack rates were shown in adults aged 18–39 and above 40 years, 2.14% (17/791) and 1.77% (14/791), respectively. Although females constituted 3.16% (25/791) of the positive results, the gender difference was not significant (χ2 = 0.168, *d.f.* = 1, p = 0.4).

**Table 3:** Distribution of Dengue IgM Seropositive cases among age groups and gender.

Age group	Number of positives/Total tested (%)		Grand total (%)
	Female (%)	Male (%)	
≤5 yrs	2/20 (10%)	1/38 (2.63%)	3/58 (5.17%)
6–17 yrs	3/90 (3.33%)	4/103 (3.88%)	7/193 (3.6%)
18–39 yrs	13/281 (4.63%)	4/88 (4.55%)	17/369 (4.6%)
40–60 yrs	6/92 (6.52%)	4/35 (11.43%)	10/127 (7.87%)
≥60 yrs	1/23 (4.35%)	3/21 (14.29%)	4/44 (9.09%)
Grand Total	25/506 (4.94%)	16/285 (5.61%)	41/791 (5.18%)

### Dengue reported cases

A total of 193 dengue cases were reported from the selected neighborhoods. Among these reported cases, 69% were males (133/193) compared to 21% females (60/193). This gender difference in attack rate was significant (λ^2^ = 5.1, *d.f*. = 1, p = 0.02). Another significant difference on attack rate was shown between the age groups (λ^2^ = 19.6, *d.f.* = 4, p = 0.0005). Accordingly, while 42% (83/193) of the reported cases ranged between (18–39) years old, 40% (77/193) were ranged (6–17) years old.

### Spatial pattern of dengue in Port Sudan city

#### Entomological indices (Container Index (CI), Breteau Index (BI), House Index (HI), Pupae/Person (P/P) and Pupae/Children (P/C))

Entomological indices of dengue in the three neighborhoods' strata are shown in Table 4. BI, HI and P/P indices were lower in the upper class neighborhoods (65, 51 and .24), as compared to the other two strata (91, 66 and .38, respectively). Overall, significant differences was shown between the upper class neighborhoods and the middle and lower strata, on CI (F = 309, *d.f*. = 2277, p<0.001), BI (F = 1872, *d.f*. = 2277, p<0.001), HI (F = 1884, *d.f.* = 1745, p<0.001), P/P (F = 3067, *d.f.* = 4870, p<0.001) and P/C (F = 2347, *d.f.* = 4870, p<0.001).

**Table 4:** Averages of *Stegomyia* [Container (CI), Breteau (BI) and House (HI)] and Pupal indices [Pupae/Person (P/P), and Pupae/Children<5 years (P/C)] of the three strata of Port Sudanduring the period November 2008–October 2009 [Confidence Intervals].

Neighborhood strata	CI	BI	HI	P/P	P/C
**Upper class**	41%*** ^(a)^_[40.5–41.5]_	65*** ^(a)^_[64.1–65.9]_	51%*** ^(a)^_[50.4–51.6]_	0.24*** ^(a)^_[0.236–0.244]_	0.93*** ^(a)^_[0.918–0.942]_
**Middle class**	53%*** ^(b)^_[52.0–54.0]_	83*** ^(b)^_[82.9–83.1]_	68%*** ^(b)^_[67.9–68.2]_	0.28*** ^(b)^_[0.277–0.283]_	1.01*** ^(b)^_[1.001–1.019]_
**Low class**	52%*** ^(b)^_[51.6–52.4]_	91*** ^(c)^_[90.4–91.6]_	66%*** ^(c)^_[65.6–66.4]_	0.38*** ^(c)^_[0.379–0.381]_	1.35*** ^(c)^_[1.343–1.357]_

*** : p<0.001 using ANOVA test.

#### Dengue incidence rate

Overall, an incidence rate of 35 new cases in 10,000 (193/54,886) people was shown (Table 5). A significant difference between the high stratum and the other two strata was shown on incidence rates (F = 73.97, *d.f*. = 2, p<0.001), 0.0031 versus 0.0036 each, respectively.

**Table 5:** IgM Seroprevalence rates compared to incidence rates in the study areas during the period November 08–Oct 09.

Neighborhood strata	Area	IgM Seroprevalence %	Incidence rate
**Upper class**	*Abuhasheish*	7.0% (7/100)	0.0032 (9/2772)
	*Down town*	4.3% (4/94)	0.0032 (10/3133)
	*Elthora*	5.6% (4/71)	0.0029 (11/3745)
	**Average/total**	**5.7% (15/265)**	**0.0031** *** **(30/9651)**
**Middle class**	*Dar Elnaeem*	3.0% (3/100)	0.0035 (21/5958)
	*Elmatar*	4.0% (4/99)	0.0038 (22/5768)
	*Salalab East*	7.8% (5/64)	0.0034 (34/9900)
	**Average/total**	**4.6% (12/263)**	**0.0036** *** **(77/21626)**
**Lower class**	Dar Elsalam	3.5% (4/113)	0.0034 (20/5931)
	Elgadisia	5.3% (4/75)	0.0036 (37/10352)
	Elwihda	8.0% (6/75)	0.0040 (29/7326)
	**Average/total**	**5.3% (14/263)**	**0.0036** *** **(86/23609)**
**Grand Total**		**5.2% (41/791)**	**0.0035 (193/54886)**

*** **: p<0.001.**

### Temporal pattern of dengue in Port Sudan

#### Entomological indices (Pupal and Stegomyia indices)

The dengue vector was found throughout the year in Port Sudan, except in September when no larvae or pupae *Ae. aegypti* was found in the inspected water containers. It is possible that *Ae. aegypti* dwells in different breeding containers during September and/or there are desiccated eggs of the mosquito in the breeding containers.

**Figure 3:**
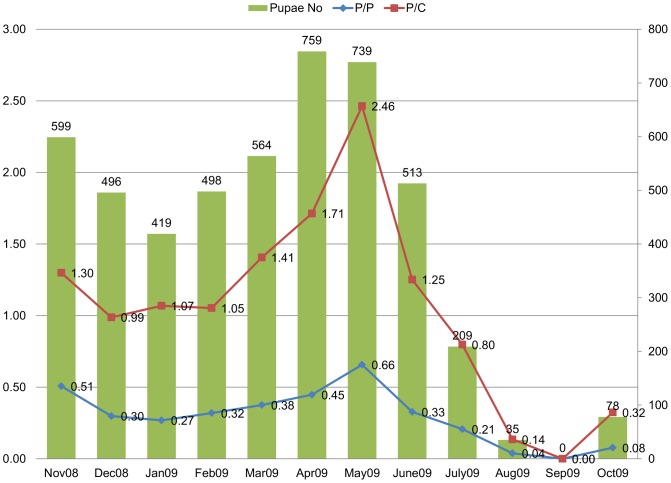
Monthly total numbers of pupae of *Aedes aegypti*, P/D and P/C indices in Port Sudan. Two peaks of pupae indices of Dengue vector: pupae/person Index (P/P) and pupae per Children<5 yrs (P/C), are shown for November and April–May during the period (November 2008–October 2009).

The largest number of pupae was collected during April (n = 759), May (n = 739) and November (n = 599). The highest P/P was shown in May (2.46) while the lowest one was in September (0). Similarly, the highest P/C was also shown in May (0.66) while the lowest one was in September (0) (see figure 3).

**Figure 4:**
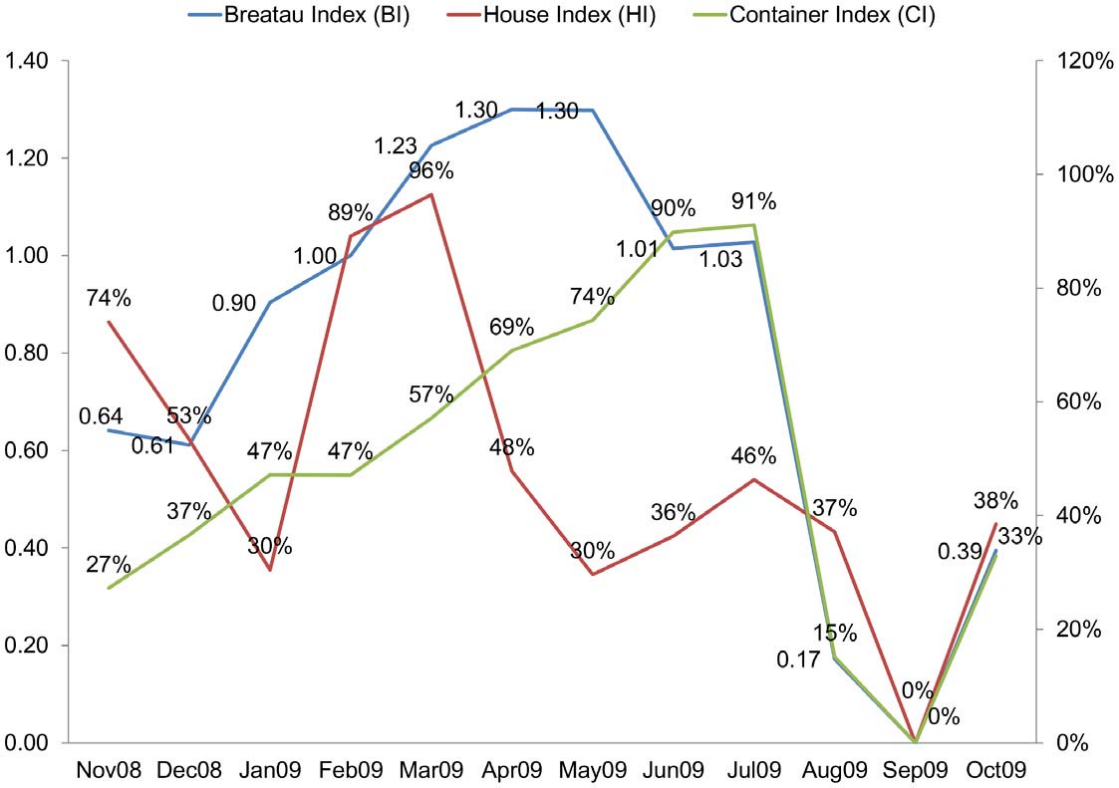
Seasonality of *Stegomyia* indices in Port Sudan. The highest *Stegomyia* indices of Dengue vector, HI, BI and CI was shown for March, April–May and July during the period (November 2008–October 2009).

**Figure 5:**
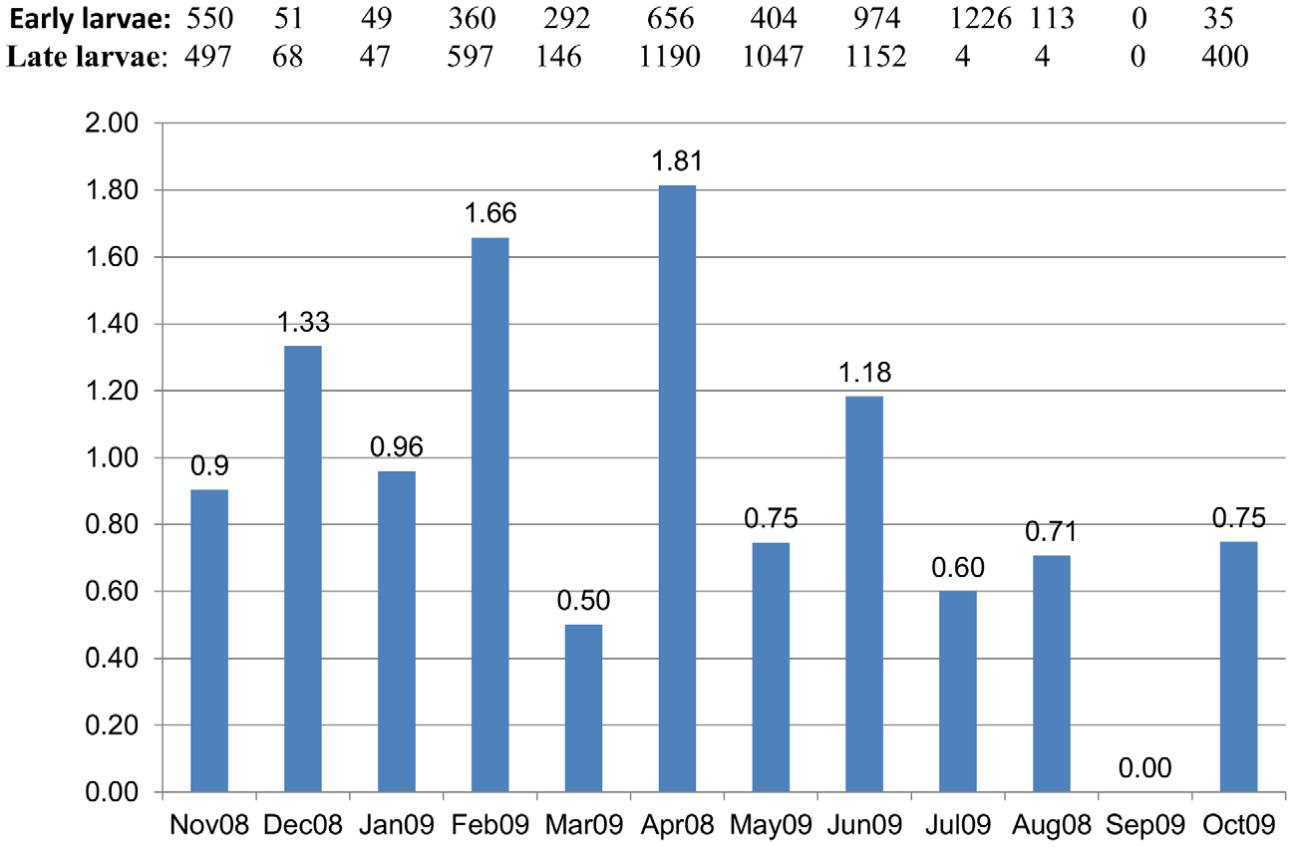
Monthly larval ratio (late/early stages) of *Ae. aegypti* in Port Sudan city. Numbers and ratios of early stages (1st/2nd) and late stages (3rd/4th) of the dengue vector collected.

The highest HI was in March (96%) while the lowest in January and May (30%). For the other two *stegomyia* indices: the highest BI was in April and May (1.3) while the lowest occurred in August (0.17). The highest CI was in July (91%) while the lowest occurred in September (0%) (see Figure 4). Larval ratio: the largest number of early larvae (i.e. 1^st^ and 2^nd^ instars) of *Ae. aegypti* was collected in July (1126), while the largest number of late larvae (i.e. 3^rd^ and 4^th^ instars) was collected in April (1190). The highest larval ratio (number of late larvae/early larvae) was 1.81 followed by 1.66 recorded in April and February, respectively (Figure 5).

### Correlation of entomological indices, meteorological parameters and seroprevalence rates

#### P/P index and IgM seroprevalence

**Figure 6:**
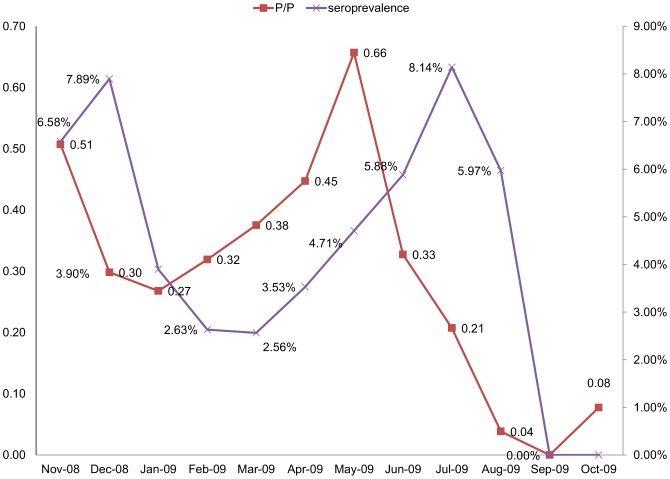
Association of IgM seroprevalence and entomological indices. Monthly patterns of IgM seroprelavence and Pupal/Demographic indices of Dengue in Port Sudan city during the period November 2008–October 2009.

Monthly curves of Pupal/Demographic index and IgM seroprevalence rates of dengue are shown in Figure 6. The M-shaped curves have two peaks. The first peak of P/P (0.51) occured in November followed by a serological peak of IgM in December (7.89%). The second and largest peak of IgM was in July (8.14%). It was preceded by a higher peak of P/P two months earlier in May (0.66) and by the highest peak of larval ration in April (1.81). Both the very low P/P in August (0.04) and disappearance of dengue vector in September resulted in zero IgM seropositivity in September and October. There is a significant correlation between P/P index and IgM sero-prevalence in the next month (Pearson *r* = 0.71, n = 11, p = 0.015).

#### Meteorological parameters and IgM seroprevalence

**Figure 7:**
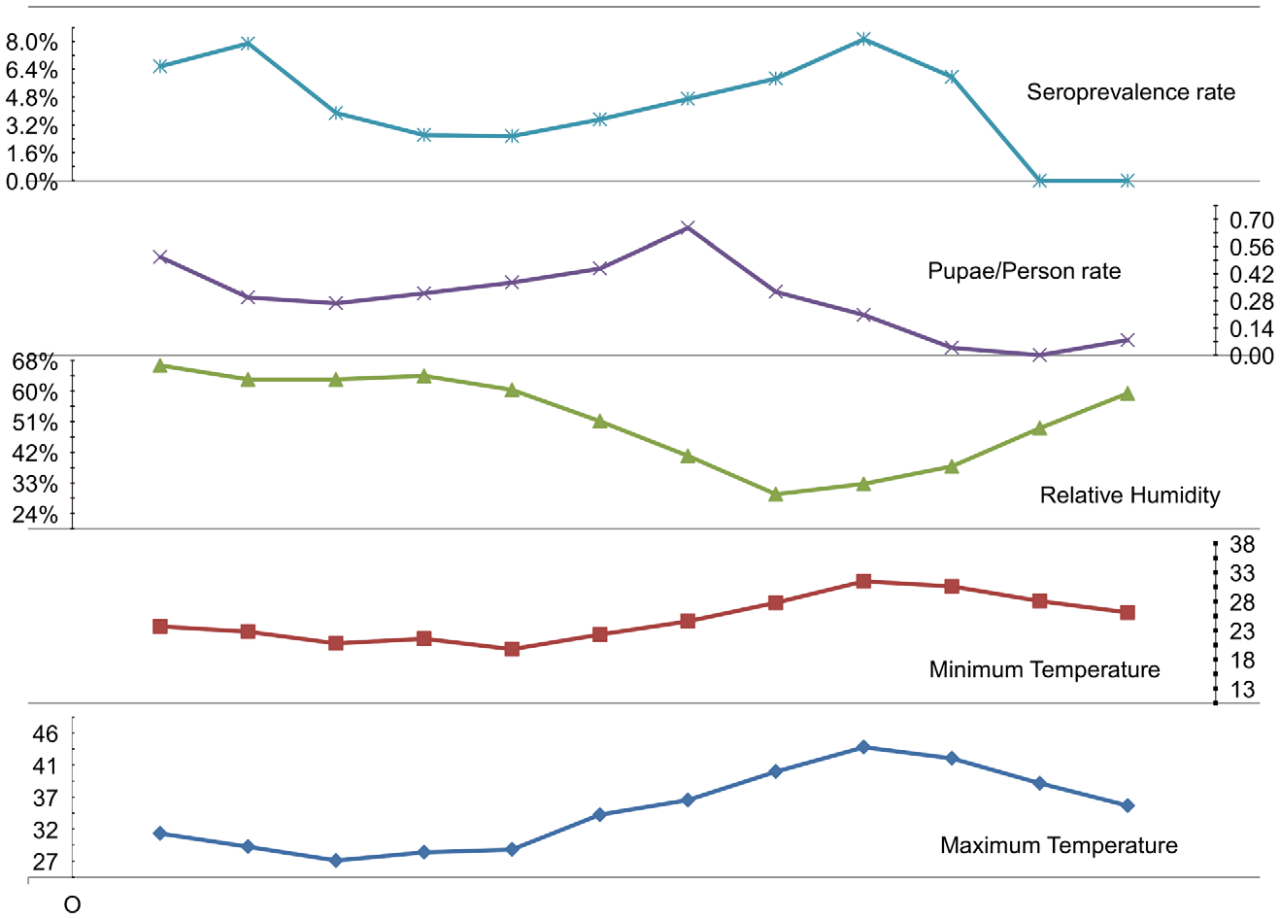
Association of meteorological parameters and IgM seroprevalence. Monthly patterns of meteorological parameters (minimum and maximum temperature and relative humidity), Pupae/person rate and IgM seroprelavence of dengue in Port Sudan city during the period November 2008–October 2009.

The two serological peaks of IgM in July and December and were preceded by the lowest and highest relative humidity during the previous months June and November (30% and 67%), respectively (Figure 7). Also, the highest monthly minimum temperature in July (31°C) was coincided with the peak of IgM seroprevalence (8.14%). Excluding months of zero prevalence, the correlation between the minimum temperature and seropositivity rates was significant (Pearson r = 0.67, p = 0.03, n = 10).

#### Meteorological parameters and entomological indices

An abrupt decrease in the relative humidity from March to June (60% to 30%) was recorded (Figure 7). This coincided with a dramatic drop in House Index (HI) from 96% to 36% during the same period. A temperature above 40°C, which continued from June to August, coincided with a sharp decline in Pupal/Demographic Index (P/P) from 0.66 in May to zero in September. The negative association between the minimum temperature and P/P index was significant (Pearson correlation = −0.83, n = 11, p = 0.027). Similarly, the maximum temperature had a negative correlation with HI (Pearson correlation = −0.65, n = 11, p = 0.032). However, the correlation was noticeable but not significant for RH% with either P/P (Pearson correlation = 0.5, n = 11, p = 0.12) or HI (Pearson correlation = 0.54, n = 11, p = 0.09).

## Discussion

This study shows dengue transmission in Port Sudan is autochthonous and related to storage of drinking water. Drinking water in Port Sudan is mainly sourced from *Khor Arbaat*, i.e. a seasonal stream or Wadi. There is a reservoir in *Khor Arbaat* about 20 km north of Port Sudan [23]. Drinking water is either pumped through pipelines or transported via motorized tankers to the city. Also, there are two desalinization facilities in the city. However, all these water sources are insufficient and supply only one third of the needed drinking water [24].

The main containers for indoor breeding of dengue vector in Port Sudan were clay pots and barrels. Owing to shortages of drinking water, the residents of Port Sudan usually preserve drinking water in these containers in close proximity to their houses. A common factor in the emergence of dengue in urban settings in developing countries is a lack of basic services for economically marginalized and growing populations [6], [25].

Both IgM seroprevalence (ranged between 3%–8% among the healthy residents) and incidence rate (35 new clinical cases per 10,000 individuals) reveals that dengue is a considerable burden on the population of Port Sudan. Similarly, a recent study in the city found a 7% IgM seroprevalence rate among pregnant women [15]. No virus serotype(s) has been determined in the current work. Co-circulation of DEN-1 and DEN-2 was confirmed in Port Sudan in 1984 [14]. In this hospital study, about 72% of the symptomatic patients were males and their ages averaged 28 years, ranging between 12–70 years. Introduction of DEN-3 in Port Sudan was confirmed during the 2004/2005 outbreak [16]. In Central Brazil, co-circulation of the three serotypes was shown [26]. Females were more affected and about 85% of the infected individuals were adults ranging between 20–70 years.

Thus, individuals of working age (18–60 years old) appeared to be more vulnerable to dengue transmission than other age groups, either because this group is subject to a secondary infection of dengue or more susceptible after a few years of transmission. Further work is needed to quantify the economic burden of dengue on the community of Port Sudan.

Dengue has an uneven spatial distribution in Port Sudan. Although lower and middle class neighborhoods have low consumption rates of drinking water compared to upper class neighborhoods, such neighborhoods have higher entomological density indices than the latter. This may be due to the large number of small containers such as clay pots utilized in the lower and middle class neighborhoods. This is in line with our finding that the incidence rate of clinical dengue is low in the middle and lower class neighborhoods. Perhaps the low and middle class neighborhoods were affected first and have higher rates of herd immunity. However, this was not supported here by a significant difference on IgM prevalence between the three study strata. Therefore, it may be linked with health seeking behavior in these economically distinct areas.

The temporal pattern of dengue in Port Sudan showed a bi-cyclic trend. Hence, the city was likely subject to two transmission peaks: the first short peak in the winter (i.e. extending for 2 months in November and December), and a second long peak in the summer (i.e. 3 months from June to August). These two peaks were preceded by peaks of mosquito densities in December and June. The current work confirms historical records of *Ae. aegypti* as the principle vector of dengue in Port Sudan [17]. No reports on invasion of *Ae. albopictus* in the city from sea ports of South East Asia was traced.

However, the disappearance of the dengue vector (*Ae. aegypti*) in September demands further research to define whether there is a true disappearance and if so how and from where the vector is reintroduced.

The coastlines of the Red Sea are subject to two monsoons: a northeasterly winter monsoon (October–April) and southwesterly summer one (May–September) [27]. The maximum temperature recorded in July and August preceded the observed crash of the *Aedes* population in August and September. However, further research is needed to determine if there is a relationship between dengue outbreaks and climate variability in the Red sea region.

In conclusion, there are spatiotemporal patterns of dengue transmission in Port Sudan. Hence it should be feasible to carry out timely vector control measures to prevent or reduce dengue transmission in this coastline area. Coastlines of the Red Sea face similar situations of insufficient drinking water and the zone is prone to dengue epidemics. Climatic variability and increased shipping traffic along the Red Sea ports (trade with China, South East Asia and Latin America) during the last decade may be key drivers for dengue outbreaks. Further research is needed to study the impact of climatic and socioeconomic changes on emergence of dengue in the Red Sea region.
